# Mentalization based treatment for a broad range of personality disorders: a naturalistic study

**DOI:** 10.1186/s12888-024-05865-2

**Published:** 2024-06-07

**Authors:** Endang Rizzi, Jonas Gijs Weijers, Coriene ten Kate, Jean-Paul Selten

**Affiliations:** 1 Rivierduinen Institute for Mental Health Care, Sandifortdreef 19, Leiden, 2333ZZ the Netherlands; 2 School for Mental Health and Neuroscience, University of Maastricht, Universiteitssingel 40, Maastricht, 6229 ER The Netherlands

**Keywords:** Mentalization-based treatment, Naturalistic study, Personality disorder

## Abstract

**Background:**

Several studies have observed that mentalization-based treatment (MBT) is an effective treatment for borderline personality disorder (BPD), but its effectiveness for other personality disorders (PDs) has hardly been examined. Additionally, the evidence supporting the claim that MBT improves mentalizing capacity is scarce. The present study examined whether (i) patients with a broad range of PDs enrolled in an MBT program would improve on several outcome measures (ii) mentalizing capacity would improve over time; (iii) patients with BPD would improve more than those with non-borderline PDs.

**Method:**

Personality disorders, psychiatric symptoms, social functioning, maladaptive personality functioning and mentalizing capacity were measured in a group of individuals with various PDs (*n* = 46) that received MBT. Assessments were made at baseline and after 6, 12, and 18 months of treatment. The severity of psychiatric symptoms, measured using the Outcome Questionnaire 45, was the primary outcome variable.

**Results:**

Overall, enrollment in the MBT program was associated with a decrease in psychiatric symptoms and an improvement of personality functioning, social functioning for a mixed group of PDs (all p’s $\le$ .01). Bigger effect sizes were observed for BPD patients (*n* = 25) than for patients with non-BPD (*n* = 21), but the difference failed to reach statistical significance (*p* = 0.06). A primary analysis showed that the decrease in psychiatric symptoms was significant in BPD patients (*p* = 0.01), not in non-BPD (*p* = 0.19) patients. However, a sufficiently powered secondary analysis with imputed data showed that non-BPD patients reported a significant decrease in psychiatric symptoms too (*p* = 0.01). Mentalizing capacity of the whole group improved over time (*d* = .68 on the Toronto Alexithymia Scale and 1.46 on the Social Cognition and Object Relations System).

**Discussion:**

These results suggest that MBT coincides with symptomatic and functional improvement across a broad range of PDs and shows that MBT is associated with improvements in mentalizing capacity. As the study is not experimental in design, we cannot make causal claims.

**Conclusion:**

Mentalization-based treatment may be an effective treatment for patients with a broad range of PDs.

**Trial registration:**

The study design was approved by the Leiden University Ethical Committee.

## Introduction

Personality disorders (PDs) comprise a category of mental health conditions characterized by enduring patterns of thinking, feeling, and behaving that significantly deviate from cultural expectations [1]. These patterns are pervasive, inflexible, and lead to significant distress or functional impairment, greatly affecting quality of life in individual patients [2]. The various types of PDs can be subdivided into three clusters, A, B, and C. Cluster A comprises the paranoid personality disorder (PPD), schizoid personality disorder (SzPD), and schizotypal personality disorder (StPD), characterized by strange and eccentric behavioral patterns. Cluster B covers the borderline personality disorder (BPD), antisocial personality disorder (ASPD), narcissistic personality disorder (NPD), and histrionic personality disorder (HPD)This cluster is marked by dramatic or impulsive behavioral patterns. Cluster C encloses the avoiding personality disorder (AvPD), dependent personality disorder (DPD), and obsessive–compulsive personality disorder (OCPD), which are defined by anxious and avoiding behavioral patterns [1].

PDs are highly prevalent, with 31–45% of psychiatric patients and 10–15% of the general adult population meeting criteria for at least one PD [3, 4]. PDs not only pose a significant psychological burden to individual patient [2], but a large economic burden to societies as well [5]. However, in spite of the large burden that PDs pose, little research has been conducted pertaining to their effective treatment. A recent meta-analysis [6] provided an overview of effective treatments for a wide range of PDs. Only five controlled studies were included in the meta-analysis that examined the effectiveness of treatments for cluster C PDs. The meta-analysis concluded that cognitive behavioral therapy seems to have a positive effect on symptom reduction. Not a single RCT had been conducted regarding the treatment of cluster A PDs. With 19 randomized controlled trials (RCTs), Borderline PD (BPD) is the most researched PD in terms of treatment [6]. There are several evidence-based treatments for BPD such as Dialectic Behavioral Therapy (e.g. 7), Schema-Focused Therapy (e.g., 8), Transference-Focused Psychotherapy [9], and the currently most investigated treatment: Mentalization-Based Treatment (MBT; 10), the subject of this study.

MBT focuses on enhancing an individual's capacity to mentalize, or the ability to reflect on mental states as they occur, particularly in the context of emotional relationships. Mentalizing is the imaginative process through which an individual interprets behavior of himself and others in terms of intentional mental states such as desires, needs, feelings, and beliefs [10]. MBT has emerged as a well-established and empirically supported therapeutic approach for individuals diagnosed with BPD. Bateman and Fonagy [10] demonstrated that MBT significantly reduces hospitalization rates, self-harm incidents, and improved social and interpersonal functioning in BPD patients. Later studies also established that these improvements remained robust, years after termination of treatment [11, 12].

Bateman and Fonagy [10] initially developed MBT for patients with BPD. However, given the ubiquitous presence of mentalizing deficits in PDs [13, 14], the therapeutic process of MBT is likely to be applicable to patients with other PDs [15]. Preliminary studies and clinical observations suggest promising results, indicating the need for further research to elucidate the full extent of MBT's applicability across various personality pathologies. Although no randomized controlled studies exist of the effectiveness of MBT for other PDs, several theoretical papers and case studies have been written regarding the implementation of MBT for ASPD [16, 17], NPD [18, 19], AvPD [20] and cluster A PDs [21, 22]. A few empirical studies have also given some support for the potential effectiveness of MBT for other PDs. Bateman et al. [23] explored MBT's effectiveness in patients with BPD and comorbid ASPD, and observed reductions in anger, hostility and paranoia. Rossouw and Fonagy [24] observed that MBT can improve mentalizing, emotional regulation and interpersonal relationships in adolescents at risk of developing PDs. Additionally, preliminary studies have shown beneficial effects of MBT on AvPD [25, 26]. Lastly a small naturalistic study has provided preliminary evidence that MBT is correlated with a long-term decrease of personality pathology and interpersonal problems in a range of PDs [27].

That MBT has garnered appeal as a treatment for a broader range of PDs may not come as a surprise, because the categorical classification of distinct PDs is controversial to begin with. The currently dominant categorical model of classification posits discrete, non-overlapping PD types, each with specific diagnostic criteria. The DSM's [1] categorical approach to diagnosing PDs has been a cornerstone of psychiatric classification; however, it faces increasing scrutiny for its rigidity and reductionism as it neglects the dimensional and dynamic nature of PDs [28, 29]. For one, there is a significant overlap in diagnostic criteria among different personality disorders, leading to high rates of comorbidity. This means that individuals often meet the criteria for multiple personality disorders simultaneously, suggesting overlapping features rather than distinct categories [30]. Furthermore, "Personality Disorder Not Otherwise Specified" (PD NOS), a diagnosis used when a patient exhibited traits of various personality disorders but did not fully meet the criteria for any one specific disorder, has been the most used category in clinical practice [31], which raises questions of the accuracy of the system in capturing the complexity and individual uniqueness of personality pathology. Lastly, the existing diagnostic framework's reliance on discrete categories fails to account for common risk factors, such as attachment-related trauma and impaired mentalizing, that contribute to the development of personality psychopathology [32].

MBT transcends the issue of category [15], as it mainly focuses on transdiagnostic, etiological factors shared by all PDs, namely: impaired mentalizing and attachment related trauma. We propose that the core components of MBT can be directly applied to treat other PDs characterized by similar underlying issues. These shared factors suggest that a treatment approach focusing on enhancing mentalization could be universally applicable and beneficial across the PD spectrum. This includes, but is not limited to, cluster A personality disorders, other cluster B disorders like ASPD and NPD, and cluster C personality disorders.

MBT may be effective at treating other PDs because of the central role it gives to attachment related trauma in the etiology and treatment of PDs. Attachment theory posits that early relationships with caregivers shape future interpersonal relationships and coping mechanisms, by providing a blueprint through which individuals engage in social interactions and the regulation of emotions [33].

The nature of early attachment interactions shapes children's expectations about relationships, including whether others can be trusted to provide comfort and whether they themselves are deserving of such comfort. These expectations, known as internal working models, in turn influencehow emotions are managed and the effectiveness of such regulation, which might involve seeking comfort or isolation [34].

Crucial for the development of a so-called secure attachment style is the presence of a reliable primary caregiver who attentively responds to the emotional needs of the infant. A secure attachment style is defined by a sense of general trust that one can and will be soothed by others, that one has a secure base from which the individual can safely explore the world [35]. Securely attached individuals also tend to seek out and maintain positive attachment relationships throughout their lives. Those with secure attachment styles have confidence in their close relationships and perceive the environment as less threatening, enabling them to cope with challenges in an adaptive, open manner. Insecure attachment styles on the other hand, are characterized by a sense of mistrust in either oneself, others, or both.

While there is just one secure attachment style, typically insecure attachment is subdivided into three categories: dismissing, preoccupied and disorganized attachment. A dismissing attachment style often develops in the context of chronic absence of support. Individuals with this attachment style tend to experience emotions less intensely and to overlook their needs for closeness, displaying compulsive self-reliance [35]. In contrast, preoccupied attachment, marked by a strong need for closeness and a higher sensitivity to potential threats and stress, leads to patterns of excessive care-seeking and dependence. This attachment style often coincides with a past of unreliable caregiver support. Meanwhile, disorganized attachment styles often developin the context of caregiver abuse where the caregiver is often perceived to be both a source of safety and a source of threat [36].

Insecure attachment is almost by definition central to the pathology of most PDs [37]. For example, ASPD, NPD, AvPD, StPD, and SzPD are often marked by a deficiency in (the quality of) attachment relationships [36]. Those afflicted with HPD and BDP are known to engage in volatile and intense interpersonal relationships [36], while individuals diagnosed with BPD and DPD are often observed to struggle with profound feelings of apprehension regarding abandonment [37]. These clinical observations have also been supported by empirical data. There is an abundance of evidence showing that insecure attachment is associated with the development of personality pathology in a broader sense [37, 38, 39, 40, 41, 42], reflecting the pervasive influence of early attachment experiences on personality development. Several studies have also found high rates of insecure attachment in individuals with specific PDs (42, 43, 44, 45, also see 46 for an overview).

Mentalization theory further posits that, next to the development of a sense of trust, insecure attachment also hampers the development of mentalizing capacity in early childhood, predisposing to psychopathology in later life [39]. According to mentalization theory, caregivers help to form a child’s mentalizing capacity by re-presenting a child’s mental states to the child [47]. In other words, this process of “symbolization” occurs through the interaction with the caregiver during childhood, emphasizing that the ability to mentalize is a developmental milestone typically nurtured within the context of a secure attachment to caregivers. In early childhood, before the capacity for mentalization has developed, children depend on their caregivers to assign meaning to their yet-to-be-understood, visceral experiences [47]. In secure attachment relationships, caregivers offer this meaning through attuned, sensitive, and somewhat exaggerated feedback to the child's sensory-affective experiences. This feedback not only helps children recognize their emotions but also teaches them that these emotional descriptors pertain specifically to their own experiences, introducing them to the concept of mentalizing by providing an external perspective on their internal states. Conversely, insecure attachment can hinder the development of an internal emotional vocabulary, potentially leading to alexithymia, i.e. difficulty in identifying and describing feelings, in adulthood [47].

According to Fonagy and Luyten [48], the process of mentalization can be described along four key axes, each with opposing poles: implicit versus explicit, cognitive versus affective, internal versus external, and self versus others. Implicit mentalization, or automatic mentalizing, occurs when mentalizing happens without conscious effort, making quick, intuitive assessments about situations or others' states. On the other hand, explicit mentalization is a more deliberate and thoughtful consideration of one's own or others' mental states [48]. The distinction between cognitive and affective mentalization lies in the methods and types of information processed. Mentalization theory posits that social understanding requires both logical reasoning about beliefs and the emotional capacity to empathize or 'feel with' another person. The differentiation between self and other in mentalization pertains to the focus of reflection, whether on one's own mental states or those of another. This differentiation should not be viewed as fixed but rather as fluid and interactive, where understanding one's emotions can inform perceptions of others, and vice versa. This aspect also includes recognizing the difference between one's own thoughts and feelings and those of others. Lastly, the internal/external divide in mentalization addresses the focus of one's reflections, whether on internal thoughts and feelings or on external attributes like appearance or actions [48].

In MBT, it is the evaluation of these imbalances and their effects on the client’s social experience, that should take center stage in evaluation and treatment [15]. As Bateman and colleagues write [15], MBT “requires the clinician to assess personality in terms of the dimensional domains of mentalizing, rather than to establish the presence of absence of specific descriptive characteristics.” In other words, it is these imbalances and each individual’s tendency towards one end of a “mentalizing axis” that needs addressing. Through the rebalancing of mentalizing, MBT aims to foster a more nuanced understanding of self and others, potentially improving social functioning and reducing harmful behaviors.

Again PDs, almost by definition, are characterized by imbalanced mentalizing. For example, patients with ASPD show a marked orientation towards cognitive mentalizing in lieu of affective mentalizing (eg. 49), i.e. they can *understand* what others may think and feel but have trouble *feeling* what others feel. Also, patients with ASPD are thoroughly oriented towards external mode of mentalizing, operating on a ‘teleological’ mode of understanding people, meaning that action is the only way to make people understand them and to understand others [15]. Helping ASPD patients to practice their ability to “imagine someone else as a human being with a separate mind” is key to reducing violence [15]. Patients with NPD are characteristically self-centered and implicit in the way they mentalize. Encouraging explicit reflection on their own and especially others' mental states, helps to rebalance the style of mentalizing towards a “we-mode” as opposed to the “me mode” [50]. On the other hand, patients with BPD find it characteristically hard to distinguish between self and other, generally referred to as disturbances in the self [51]. Such a disturbance involves an overly affect-oriented style of mentalizing as opposed to cognitive mentalizing (see 52 for an overview), meaning that they empathize easily with the feelings of others, but tend to experience difficulties to cognitively understand others or to distinguish between themselves and others when (interpersonal) emotional tension increases [51]. These patients need help regulating emotional tension in order to remain capable of cognitively distinguishing self-generated feelings and feelings generated by affective empathizing [15]. Lastly, similar to BPD, patients with AvPD have problems distinguishing self from other, resulting in emotional contagion (being to susceptible to the emotions of others [51]. In contrast to BPD, AvPD patients show a low level of reflectivity on emotional states of self and excessively focus on others’ thoughts about the self [15]. In other words, they consistently deemphasize the importance of their own emotions, while overemphasizing the perceived judgments others, making them highly sensitive to criticism. In general, AvPD patients must learn to pay attention to and learn to express their own affective experiences in the here-and-now as opposed to ruminating about potential future rejections [15].

The focus on particular mentalizing imbalances in the individual client, makes MBT highly adaptable and enhances its potential applicability to a spectrum of personality disorders. Further facilitating the treatment of a broad range of PDs is that the MBT approach to mentalizing imbalances essentially remains the same despite diagnostic category: clinicians adopt a not-knowing stance to help foster curiosity towards the client’s (often automatic) mental processes in order to detect and identify particular mentalizing difficulties [15]. The point here is to help the client detect and consciously contain non-mentalized feeling states in order to prevent them from cascading into problematic automatic patterns of behavior. Lastly, although manualized, MBT is not confined to rigid treatment steps, but rather allows for flexibility in tailoring interventions to the unique needs of the individual [15]. This should make MBT applicable to a range of PDs.

However, although theory and clinical experience suggest that MBT may be suitable for a wider range of PDs, empirical evidence supporting this claim remains scarce. Only one, rather under-powered, study has provided preliminary evidence that MBT is related to a decrease of symptoms in a range of PDs [27]. Additionally, while mentalization theory posits that MBT works through the improvement of mentalizing capacity, only a few studies thus far have shown that MBT actually increases mentalizing capacity [67, 53, 54]. The current study therefore examines a) whether patients with various PDs who receive MBT improve over time on a range of outcomes (and whether this improvement is significantly different in BPD as opposed to other PDs) and b) whether mentalizing capacity improves as well.

## Methods

### Study design

The study was of a longitudinal, uncontrolled design. Patients with a PD and receiving MBT were followed during their treatment. Mentalizing-based treatment had a maximum duration of 18 months. All participants were given the same information during the process in the form of a letter. The letter detailed what participating in the study entailed (time-investment, length of the study, participation incentives), the right to decline participation without consequences and how the anonymity of and security of participant data was handled.

### Participants

The study was conducted at the Dutch mental health institute Rivierduinen, which offers outpatient and inpatient care for people with severe and complex psychiatric disorders.

Patients diagnosed with a PD and indicated for MBT were asked to participate in the study before the start of treatment. The coordinating investigator (ER) provided verbal and written information. All participants gave informed consent, no patient declined. If patients had declined participation, they would not have been disadvantaged by their decision and would have remained eligible for MBT.

Eligible participants were adult men and women diagnosed with a PD (based on Structured Clinical Interview for DSM-IV Axis II Personality Disorders (SCID-II) or International Personality Disorder Examination (IPDE) and having psychiatric symptoms such as anxiety and depression. Individuals with a co-morbid psychotic disorder, insufficient understanding of the Dutch language, or substance abuse that necessitated treatment were excluded.

Using the G*Power software, a power analysis was conducted to determine the required sample size. To achieve a power of > 0.80 for a dependent t-test (repeated measures) with an α level of 0.05 and an effect size of 0.5, 45 participants are needed. Factoring in the typical dropout rate of 25%, the total sample size required is (45 + 11.25) 56.25, which rounds up to 57 participants.

### Structure of treatment program

Mentalization-based treatment is a manualized, outpatient, psychodynamic treatment program. At the Rivierduinen mental health care center, two variants were provided: [1] twice a week group therapy, once a week individual psychotherapy, and once a week non-verbal therapy (sensorimotor therapy); and [2] once a week group therapy and once a week individual psychotherapy. Indication for treatment condition 1 or 2 was made based on the severity of symptoms and social functioning. A detailed description of the MBT principles, interventions, and program components are beyond the scope of the present paper but are provided in Allen et al. [55] and Bales et al. [56]. All the therapists were MBT-trained. Additional training was provided every two weeks by an MBT supervisor.

### Measures

Table 1 displays the outcome measures assessed at different points in time.

**Table 1:** Outcome measures taken at the different points in time

	**Baseline** **(T0)**	**6 months after start of treatment** **(T1)**	**12 months after start of treatment** **(T2)**	**18 months after start of treatment** **(T3)**
OQ-45.2	X	X	X	X
SIPP-sf	X	X	X	X
SF36	X	X	X	X
TAT	X			X
TAS-20	X			X

*OQ-45.2* Outcome Questionnaire, *SIPP-SF* Severity Indices Personality Problems-Short Form, *SF36* Short Form 36, *TAT* Thematic Apperception Test, *TAS-20* Toronto Alexithymia Scale-20

Treatment outcome was measured at the start of treatment (T0), at 6 months (T1), 12 months (T2), and 18 months (T3), in the areas corresponding to the treatment goals (i.e., (1) psychiatric symptoms, (2) social and interpersonal functioning, (3) personality functioning, and (4) mentalizing capacity. Assessments were conducted by an independent researcher or by an independent research assistant, not involved with treatment. The outcomes were not communicated to the therapists nor to the participants during the course of the study. At the end, the participants received information on the conclusions at group level.

### Primary outcome

The primary outcome was general psychiatric symptoms measured by the Outcome Questionnaire 45.2 (OQ-45.2) [57], which includes three subscales: subjective discomfort (intra-psychological functioning), (dis)functioning in interpersonal relationships, and (dis)functioning in the social role. The OQ-45.2, a self-report instrument includes 45 items that were scored on a 5-point scale from 0 (never) to 4 (always). This resulted in a total score from 0 to 180. A higher score reflects more problems. A separate score per domain could also be obtained. The psychometric characteristics of the OQ-45.2 are adequate [57].

### Secondary outcome

Maladaptive personality traits were measured using the Severity Indices for Personality Problems-short form (SIPP-SF; 58). The test subject indicates on a 4-point scale to which extent the 60 items have been applicable in the last three months. The items are clustered in five domains: (1) self-control—impulse control and the ability to tolerate and control emotions (emotion regulation); (2) Identity integration—stable, integrated and positive self-presentation, and consider one’s own life as meaningful; (3) Responsibility—the ability to set realistic goals, internalize external norms and values, and to live by them; (4) Relational functioning—the ability to enter and maintain long-lasting intimate relationships; (5) Social concordance—the ability to treat others in an equivalent and respectful way. The psychometric qualities are sufficient [59].

General health was measured using the Short Form 36 (SF36;60). The questionnaire consists of eight subscales that include physical functioning, physical and mental role functioning, social functioning, mental functioning, vitality, pain, and experiencing health.

The SF36 consists of 36 questions. The answer type changes per subscale, with some using yes/no answers and others 3-, 5-, or 6–point Likert scales. The higher the score, the more problems the person experienced in that aspect. The psychometric qualities of the Dutch translations have sufficient psychometric properties [61].

### Measuring mentalizing capacity

The Thematic Apperception Test (TAT; 62), scored with the Social Cognition and Object Relation Scale (SCORS) of Westen [63], assesses four dimensions of social cognitive, i.e. mentalizing, capacity: the complexity of the mental representations of people, understanding social causality, the affect-tone of relationships, and the capacity for emotional investment. Each dimension is scored on a 5-point scale. A higher score indicates higher social cognitive functioning. Six pictures from the TAT were used. The responses of the test subjects were recorded on tape and transcribed verbatim. Responses to the TAT, analyzed with the SCORS, are a valid and reliable way to measure social cognition 64; 65), with good internal consistency between pictures [65] and good [65] to excellent [64] inter-rater reliability. The TAT is one of the few instruments that measures multiple domains of the mentalizing construct, including affective and cognitive aspects [66]. In the current study, raters were blinded with regard to the time point of the TAT responses. Interrater reliability was assessed by means of recorded narratives and rated independently by all raters, which included one of the authors and students with a bachelor’s degree in psychology. Inter-rater reliability was acceptable for complexity of representations and understanding of social causality (ICC = 0.7), good for affect-tone of relationships (ICC = 0.8), and excellent for capacity for emotional investment (ICC = 0.9).

The Toronto Alexithymia Scale-20 (TAS-20; 67) measures self-rated alexithymia. It comprises 20 items measured on a 5-point scale divided into three subscales: difficulty identifying feelings, difficulty expressing feelings, and externally oriented thinking. The higher the score, the more problems were experienced on the relevant subscale. The psychometric qualities for the internal reliability and validity are sufficient [68]. The test–retest reliability has been reported as 0.77 [69].

### Statistical analysis

#### Primary analyses

All analyses were carried out with IBM SPSS Statistics for Windows (version 24). The data analysis was carried out according to the intention to treat principle. A multilevel analysis (linear mixed model) was used to evaluate the changes over time (T0, T1, T2, and T3) on the primary and secondary outcome measures (OQ-45–2, SIPP-SF, SF-36). Time was treated as a linear factor, which implies that the regression coefficients measure the change from the start of treatment until the end of treatment. The independent variable was type of PD, and the dependent variables were psychiatric symptoms and social and interpersonal functioning. The model was based on random intercepts and fixed slopes.

Effect sizes were calculated using Cohen’s *d* [70]. The average scores on the outcome measures on baseline (T0), T1, T2, and T3 were compared. Cohen's *d* was calculated for the psychiatric symptoms (OQ-45.2, subscale intrapsychic functioning), social functioning (OQ-45.2, subscale interpersonal relationships), personality functioning (SIPP-SF), general health (SF36), and the mentalizing capacity (TAS-20, TAT dimensions).

#### Moderation analysis

An additional moderation analysis was conducted to analyze whether type of diagnosis (either Borderline or other personality disorders) was associated with change over time in overall personality functioning. Personality Disorder-type was dichotomized with either BPD (= 0) or other PDs (= 1). A repeated measures ANOVA was used to evaluate the changes over time on the primary OQ-45–2. The independent variable was time (T0, T1, T2, and T3), with OQ-45–2 as dependent variable and type of PD as between-subject variable.

#### Handling missing data

Missing data due to nonresponse was substantial and varied over time (from 37% at T1, 50% at T2, and 35% at T3), but was consistent with previous studies (see 71 for an overview). As missing data can lead to an increase of both type I and type II errors, a secondary analysis was conducted using multiple imputation [72] to gauge the effects of missingness. Because drop-out was unlikely to have been random, independent variables were used that were previously reported to predict drop-out in the treatment of personality disorders, including gender, age, severity of symptoms [73], level of education [74], and mentalizing capacity [75] at T0. Additionally for obvious reasons the intensity of treatment program (either one, two or three days a week) was included. For each analysis, five imputed datasets were created using a fully conditional Markov chain Monte Carlo approach. Results from analyses conducted with the imputed datasets were combined using Rubin’s rules. Only the analysis of the primary outcome variable (OQ-45–2) and the moderation analysis received this treatment. Again, a general linear repeated measures analysis was used to evaluate the changes over time on the primary OQ-45–2. The independent variable was time (T0, T1, T2, and T3), with OQ-45–2 as dependent variable and type of PD as between-subject variable.

#### Correcting for multiple testing

According to the European Medicines Agency guidelines [76], dividing outcomes into primary and secondary outcomes is a way to control for Type I error rate. However, in this way, secondary outcomes can only be considered as indications—not evidence—of potential treatment effects.

## Results

### Participant adherence

**Fig. 1:**
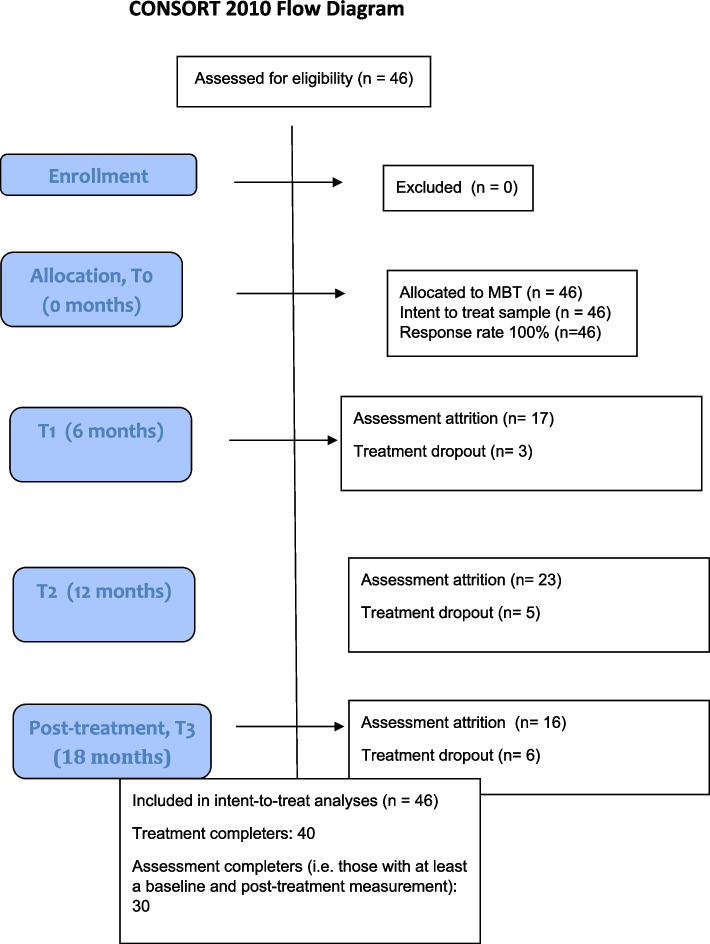
CONSORT flow diagram of patient progression through the MBT program and response rate to primary outcome self-report

The patient flow is presented in Fig. 1. All participants were included in the statistical analysis.

### Patient characteristics

For patient characteristic please refer to Table 2 below.

**Table 2:** Characteristics of the sample

Characteristics at baseline	
Female, *n/N* (%)	31/46 (67.4)
Age, years, mean (*SD*)	31.6 (9.5)
Highest level of education, *n/N* (%)	
primary school	1/46 (2.2)
Completed high school	16/46 (34.8)
Some additional education/training	16/46 (34.8)
Completed undergraduate	11/46 (23.9)
Completed postgraduate	2/46 (4.3)
Comorbid psychiatric diagnosis, *n/N* (%)	
Depressive disorder	16/46 (34.8)
Post-traumatic stress disorder	5/46 (10.9)
Anxiety disorder	0
Substance abuse disorder	6/46 (13)
Eating disorder	2/46 (4.3)
Attention Deficit Hyperactivity Disorder	4/46 (8.7)
Autism	1/46 (2.2)
None	12/46 (26.1)
Personality diagnosis, *n/N* (%)	
Paranoid PD	1/46 (2.2)
Schizoid PD	0
Schizotypal PD	0
Histrionic PD	0
Narcissistic PD	1/46 (2.2)
Borderline PD	25/46 (54.3)
Antisocial PD	0
Avoidant PD	10/46 (21.7)
Dependent PD	0
Obsessive–Compulsive PD	0
PD Not Otherwise Specified	9/46 (19.6)
Comorbid PD, *n/N* (%)	
None	34/46 (73.9)
Paranoid PD	1/46 (2.2)
Narcissistic PD	1/46 (2.2)
Borderline PD	3/46 (6.5)
Antisocial PD	1/46 (2.2)
Avoidant PD	4/46 (8.7)
Dependent PD	1/46 (2.2)
Obsessive–compulsive PD	1/46 (2.2)

*PD* Personality Disorder, *PSTD* Post-Traumatic Stress Disorder, *ADHD* attention deficit hyperactivity disorder

### Treatment dropout

Patients who completed the therapy (*N* = 40; 87%) had been treated for an average of 14.0 months (*SD* = 5.6), while dropouts (*N* = 9) had been treated for an average of 5.7 months (*SD* = 3.6). Reasons for dropout were—in one case—suicide, and in the other cases increase of alcohol and/or drug abuse that needed treatment, getting a job, and a decrease in motivation for the therapy. The primary and secondary outcome measures did not significantly differ between patients who completed the treatment and those who did not. There was also no significant difference in the type of PD, co-morbid psychiatric diagnosis, age, gender, and level of education in the baseline results of dropouts and those who finished the treatment. Lastly there were no significant differences at baseline on the primary and secondary outcome measures between patients who did and did not complete participation in the study.

### Changes in symptomatic distress, interpersonal, social, and personality functioning, and general health during treatment

Observed means and standard deviations for all four measurement points are presented in Table 3.

**Table 3:** Means, standard deviations, multilevel model of change: symptomatic distress, social functioning, interpersonal functioning, maladaptive personality functioning, and quality of life

	**Baseline** ***N* = *46*** ***M* (*SD*)** **T0**	**6 months** ***N* = *29*** ***M* (*SD*)** **T1**	**12 months** ***N* = *23*** ***M* (*SD*)** **T2**	**18 months** ***N* = *30*** ***M* (*SD*)** **T3**	***b* (95% CI)**	***t*-test (df)**	***P* value**	**Cohen’s *d*** **(T0 vs T3)**
**Symptom questionnaire (*OQ-45.2*)**								
Total	97.87 (19.58)	88.66 (20.96)	86.66 (31.10)	75.24 (25.28)	-24.96 (-34.56 to -15.37)	*t (*23.69) = -5.37	< .001	1.00
SD	61.61 (13.06)	56.76 (16.18)	54.09 (20.17)	46.14 (17.34)	-16.98 (-23.30 to -10.65)	*t* (31.23) = -5.48	< .001	1.00
IR	21.85 (6.55)	19.07 (5.85)	20.04 (9.01)	16.93 (6.09)	-5.72 (-8.18 to -3.27)	*t* (34.25) = -4.73	< .001	.80
SR	14.41 (5.09)	12.83 (4.35)	12.78 (5.94)	12.7 (4.62)	-2.49 (-4.41 to -.57)	*t* (40.47) = -2.62	.01	.46
**Personality functioning (*SIPP-SF*)**								
SLFC	28.00 (7.06)	29.07 (8.52)	32.96 (7.54)	34.66 (6.65)	7.08 (4.43 to 9.74)	*t* (31.32) = 5.44	< .001	.97
ii	23.43 (7.08)	25.33 (6.63)	28.87 (9.68)	32.38 (9.07)	9.57 (6.24 to 12.91)	*t* (52.42) = 5.76	< .001	1.10
RESP	33.41 (6.61)	34.93 (5.51)	35.65 (6.94)	37.48 (5.76)	3.94 (1.19 to 6.07)	*t* (27.91) = 3.80	.001	.66
REL	25.93 (7.23)	24.97 (5.82)	30.07 (8.87)	27.09 (8.88)	4.67 (1.69 to 7.47)	*t* (43.64) = 3.16	.003	.14
SOC	31.50 (6.12)	32.93 (6.77)	34.57 (7.62)	35.45 (5.97)	2.63 (.69 to 4.57)	*t* (29.53) = 2.77	.01	.67
**General Health (*SF36*)**								
SF	7.02 (2.21)	6.00 (1.94)	6.35 (2.44)	5.32 (2.07)	-1.97 (-2.87 to -1.08)	*t* (29.17) = 4.53	< .001	.79
GH	12.26 (3.82)	11.87 (3.89)	11.61 (4.07)	10.86 (3.75)	-1.41 (-12.16 to -9.54)	*t* (28.53) = 1.99	.06	.37

*OQ-45.2* Outcome Questionnaire, *SD* Symptomatic Distress, *IR* Interpersonal Relation, *SR* Social Role, *SIPP-SF* Severity Indices Personality Problems-Short Form, *SLFC* Self-Control, *ii* Identity integration, *RESP* Responsibility, *REL* Relational capacities, *SOC* Social concordance, *SF36* Short Form 36, *SF* Social Functioning, *GH* General Health

#### Psychiatric symptoms

As shown in Table 3, general symptoms of distress, anxiety, and depression improved significantly during treatment. Effect sizes of the change between T0 and T3 were large.

#### Interpersonal and social functioning

Interpersonal problems, interpersonal relations and social role functioning improved during the treatment (see Table 3). Effect sizes ranged from 0.14 to 0.80, which can be interpreted as small to moderate effects.

#### Personality functioning

Maladaptive personality components associated with PDs decreased. Self-control, the ability to handle one’s own emotions and impulses in an adequate way, identity integration, responsibility, and social concordance started to improve within the first six months. Effect sizes ranged from 0.66 to 1.10, which can be interpreted as moderate to large effect sizes.

#### General health

The item general health and well-being on the SF36-MH showed no statistically significant improvement.

### Mentalizing and symptomatic distress throughout treatment

As shown in Table 4, mentalizing capacity (TAS-20, TAT-COM, TAT-EMI, and TAT-SC) increased significantly during treatment. The effect sizes varied from medium (TAS-20; TAT-COM and TAT-EMI) to large on the dimension TAT-SC. The affect-tone of relationships, measured with the TAT-AFF, did not increase during treatment.

**Table 4:** Means, standard deviations, and paired sample test

	**Baseline** **M (SD)** ***N* = 30**	**18 months** **M (*SD*)** *N*** = 30**	**Mean differences** **(SD)**	**Lower – Upper bound**	***t*-test (df)**	***p* value**	**Cohen’s *d*** **(T0 vs T3)**
TAS-20	60.03 (11.78)	52.00 (11,79)	-8.03 (11.29)	(-12.25 to -3.82)	*t* (29) = -3.90	.001	0.68
TAT-COM	2.09 (0.63)	2.47 (0.35)	0.39 (0.62)	(-0.15 to 0.62)	*t* (29) = 3.39	.002	0.75
TAT-AFF	2.89 (0.50)	2.88 (0.43)	.00 (0.58)	(-0.21 to 0.21)	*t* (29) = -0.03	.97	0.02
TAT-EMI	1.49 (0.47)	1.82 (0.37)	0.33 (0.53)	(0.14 to 0.53)	*t* (29) = 3.47	.002	0.78
TAT-SC	1.93 (0.35)	2.42 (0.32)	0.49 (0.47)	(0.32 to 0.66)	*t* (29) = 5.76	< .001	1.46

*TAS-20* Toronto Alexithymia Scale-20, *TAT-COM* TAT complexity of mental representations, *TAT-AFF* TAT affect-tone of relationships, *TAT-EMI* TAT emotional investment, *TAT-SC* understanding of social causality

### Moderation of treatment effect by type of diagnosis

Results of the moderation analysis revealed no significant interaction effect between diagnosis and time (*F* (14,3) = 3.28, *p* = 0.06), meaning that the effect of treatment was not significantly affected by the type of personality disorder (BPD or not). However, analyses per subgroup did reveal that BPD patients showed a significant decrease in symptoms over time (*F*(7,3) = 12.76, *p* = 0.01) from baseline (*M* = 102.50, *SD* = 6.72) to T3 (*M* = 68.50, *SD* = 9.58), whereas patients with another PD did not (*F* (7,3) = 1.72, *p* = 0.19; baseline *M* = 95.00, *SD* = 4.87; T3 *M* = 81.00, *SD* = 8.12).

### Secondary analyses with multiply imputed data

The percentage of imputed data differed per measurement timepoint. At baseline 0% (*N* = 0) was imputed, at T1 37% (*N* = 17), at T2 50% (*N* = 23) and at T3 30% (*N* = 16). Results with multiply imputed data again showed no significant difference between BPD and non-BPD patients, *F* (45,3) = 12.87, *p*_pooled_ = 0.07. Subgroup analyses showed that both patients with BPD (*F* (25,3) = 9.31, ${\eta }_{p}^{2}$_pooled_ = 0.56, *p*_pooled_ < 0.001) and those with other types of PD (*F* (21,3) = 5.04, ${\eta }_{p}^{2}$_pooled_ = 0.46, *p*_pooled_ = 0.01) reported a significant decrease in symptoms on the OQ-45 over time.

## Discussion

The results of this naturalistic study suggest that exposure to mentalization-based treatment coincides with a decrease in psychiatric symptoms and an improvement of personality functioning, social functioning, and mentalizing capacity for a mixed group of PDs. Effect sizes for both primary and secondary outcome measures varied from small to large. The dropout rate (19.6%) was similar to those reported in systematic reviews of treatments for PDs [77] and to those in MBT trials (10, 11, 12;; 75; 79).

The general psychiatric symptom effect size (*d* = 1.00) was greater than that found by Löf, Clinton, Kaldo, and Ryden [78] in their longitudinal, uncontrolled study of the MBT program for BPD patients, (*d* = 0.58), but comparable to that reported by Bales and colleagues [79], (*d* = 1.06)..

Results from this study also indicate that MBT improves mentalizing capacity over time, in line with previous findings [53, 54]. We observed an increase in mentalizing capacity, measured with the TAS-20 and the TAT (according to the SCORS). Our results agree with those reported by Löf and colleagues [78]. Their naturalistic study showed that self-reported alexithymia (TAS-20)which is conceptually associated with mentalizing capacity [80], decreased. This outcome suggested an increase in the capacity to identify feelings. Furthermore, we found an increase the complexity of mental representations (TAT-COM) and the understanding of social causality (TAT-SC), i.e. the cognitive aspects of mentalizing ability, and in emotional aspects of mentalizing, such as the capacity to invest emotionally in relationships (TAT-EMI). The affective tone of relationships (TAT-AFF), however, remained the same. This is similar to an earlier study that examined the effect of MBT on mentalizing capacity [54]. The latter dimension was shown to be less reliable and this may have influenced this outcome [65].

The observed effect sizes for interpersonal functioning in this study, varying between 0.14 and 0.80, were smaller than in the Bales et al. study [56], where effect sizes varied between 0.81 and 1.36. The MBT program investigated by Bales and colleagues was more intensive than that of our study. We expect that a greater effect size would have been found if our treatment had been as intensive as that used by Bales and colleagues [56]. The results also suggest that personality functioning improved during treatment. Effect sizes varied between 0.14 and 0.97. Again, the observed effect sizes in the current study were lower than those reported by Bales and colleagues [56], which varied between 1.23 and 1.74.

Lastly, while patients in the BPD subgroup showed a significant reduction in symptoms and those in the non-BPD subgroup did not, a moderation analysis revealed that the difference between diagnosis categories failed to reach statistical significance. Because power for the sub-group analysis was very low due to drop-out, a secondary moderation analysis was conducted with multiply imputed data. This analysis again revealed no significant difference between the types of patients but observed that both groups of patients showed significant reductions in symptoms over time, suggesting that power may have been lacking in the initial analysis. Thus, evidence that BPD patients benefit more from MBT than patients with other PDs is limited. Our results further suggest that the decision of providing MBT based on type of PD diagnosis may be objectionable, as the distinction does not seem clinically relevant where MBT effectiveness is concerned. Indeed, although no causal statements can be made on the basis of this study, data does support the notion that MBT may be suitable for a wider range of patients with PDs, at least with regard to AvPD and PD NOS.

### Limitations of the study and directions for future research

Despite its strengths, this study had several limitations. First, this was a naturalistic, uncontrolled study. Hence, no definitive causal claims can be made regarding MBT’s effectiveness regarding a variety of PDs. Causal claims can only be made based on experimental studies, meaning this study should be replicated with a control group as part of a randomized controlled trial. For this reason, the results should be interpreted with caution. Second, in this sample, the non-BPD group was smaller than the BPD group, which increases the chance for both type I and II error for the results of this group and thus may result in a skewed comparison. Third, since there was no follow-up measurement, the durability of the effects remain uncertain. Fourth, other variables not included in the current study should be examined for their effect on psychiatric symptoms, such as the intensity of treatment, number of comorbid disorders, the role of medication, and the role of the therapeutic relationship as the motives for dropout. Fifth, the study was impacted by a relatively high rate of attrition (although consistent with previous studies examining PDs, see 60 for an overview). As a result, the final sample was smaller than the pre-calculated power analysis called for, which may have influenced the results. Larger sample sizes tend to decrease the chance for type I and II errors; this may be particularly pertinent regarding the moderation analysis. Also, attrition may have influenced the findings due to biased nonresponse. To address this potential issue, we employed imputed data to minimize the effect of selective attrition. And although it is acknowledged that imputation may become less reliable with increasing attrition rates, recent research has demonstrated that multiple imputation can still yield acceptable results even when missing data is substantial (up to 50%), as demonstrated by Krause and colleagues [81]. Sixth, the sample of non-BPD patients primarily consisted of patients with AvPD and PD NOS, with one NPD and one PPD patient. This means that the generalizability of results to other PDs than AvPD and PD NOS is highly limited.

Despite these limitations the current study is, to the best of our knowledge, one of the few naturalistic studies that examined the effect of MBT on both personality functioning and mentalizing ability in a broad spectrum of PDs. These results lend preliminary support to the notion that participation in the MBT treatment program coincide with increases of mentalizing capacity andthat MBT could be effective for a broader range of PDs than just BPD, at least AvPD and PD NOS. However, as the study is not experimental in design, we cannot make causal claims.

## Data Availability

The datasets generated and/or analyzed during the current study are not publicly available because patients did not provide consent for their data to be shared with third parties. However, data may be available from the corresponding author upon reasonable request and with added patient consent for such data to be shared.

## Abbreviations

MBT: Mentalization based treatment
PD: Personality Disorder
AvPD: Avoidant Personality Disorder
DPD: Dependent Personality Disorder
OCPD: Obsessive Compulsive Personality Disorder
BPD: Borderline Personality Disorder
HPD: Histrionic Personlaty Disorder
ASPD: Antisocial Personality Disorder
NPD: Narcissistic Personality Disorder
StPD: Schizotypal Personality Disorder
SPD: Schizoid Personality Disorder
PPD: Paranoid Personality Disorder
PD NOS: Personality Disorder Not Otherwise Specified
RCT: Randomized Controlled Trial
TAT: Thematic Apperception Task
SCORS: Social Cognition and Object Relations System
OQ-45.2: Outcome Questionnaire
SIPP-SF: Severity Indices Personality Problems-Short Form
SF36: Short Form 36
TAS-20: Toronto Alexithymia Scale-20
